# Supplementary material for: Association of toll-like receptors single nucleotide polymorphisms with HBV and HCV infection: research status
Source: PeerJ. 2022 Apr 19;10:e13335. doi: 10.7717/peerj.13335 (PMC9029363; doi:10.7717/peerj.13335)
Supplement: Supplemental Information 1 [file peerj-10-13335-s001.docx]

| Polymorphism | Author | Year | population | Sample size | | MAF(%)  (controls) | Influence on | References |
| --- | --- | --- | --- | --- | --- | --- | --- | --- |
|  |  |  |  | cases | controls |  |  |  |
| rs3804100  (T/C) | Chen et al. | 2017 | Chinese Han male | 688 | 686 | 25.25 | HBV clearance and the risk of HBV-related HCC | ^[46]^ |
|  | Chen et al. | 2011 | Chinese Han | 24 | 46 | 22.70 | Response to hepatitis B vaccine | ^[47]^ |
| rs3804099  (T/C) | Lin et al. | 2018 | Chinese Han | 60 | 151 | 43.75 | The progression of hepatitis B | ^[42, 48]^ |
|  | Xie et al. | 2012 | Chinese | 211 | 232 | 31.68 | Susceptibility to HCC | ^[48]^ |
| rs4696480  (T/A) | Lin et al. | 2018 | Chinese Han | 60 | 151 | 23.8 | The progression of hepatitis B | ^[42]^ |
| Abbreviation: MAF: minor allele frequency; HCC: Hepatocellular carcinoma. | | | | | | | | |
