# Supplementary material for: Association of toll-like receptors single nucleotide polymorphisms with HBV and HCV infection: research status
Source: PeerJ. 2022 Apr 19;10:e13335. doi: 10.7717/peerj.13335 (PMC9029363; doi:10.7717/peerj.13335)
Supplement: Supplemental Information 2 [file peerj-10-13335-s002.docx]

| Polymorphism | Author | Year | population | Sample size | | | MAF(%)  (controls) | Influence on | References | |
| --- | --- | --- | --- | --- | --- | --- | --- | --- | --- | --- |
|  |  |  |  | cases | controls | |  |  |  |  |
| rs4986790  (A/G) | Sghaier et al. | 2019 | Tunisian | 274 | | 360 | 19.02 | Susceptibility to HBV infection and HBV-related HCC | ^[10]^ |  |
|  | Wu et al. | 2012 | Taiwanese | 278 | | - | - | HBV clearance | ^[64]^ |  |
|  | Cussigh et al. | 2013 | Caucasian | 191 | | 210 | - | The progression of HBV-related liver diseases | ^[65]^ |  |
|  | Pires-Neto et al. | 2015 | Brazilian | 121 | | 299 | 4.01 | Susceptibility to HBV infection | ^[66]^ |  |
| rs4986791 (C/T) | Pires-Neto et al. | 2015 | Brazilian | 121 | | 299 | 4.01 | Susceptibility to HBV infection | ^[66]^ |  |
| rs11536889  (G/C) | Zhou et al. | 2011 | Chinese Han | 12 | | 113 | 22.60 | HBV recurrence rate after liver transplantation | ^[67]^ |  |
|  | Zhang et al. | 2016 | Chinese Han | 949 | | - | - | The risk of HBV-related HCC | ^[68]^ |  |
| rs2149356  (A/C) | Zhang et al. | 2016 | Chinese Han | 949 | | - | - | The risk of HBV-related HCC | ^[68]^ |  |
| Abbreviation: MAF: minor allele frequency; HCC: Hepatocellular carcinoma. | | | | | | | | | |  |
