# Supplementary material for: Association of toll-like receptors single nucleotide polymorphisms with HBV and HCV infection: research status
Source: PeerJ. 2022 Apr 19;10:e13335. doi: 10.7717/peerj.13335 (PMC9029363; doi:10.7717/peerj.13335)
Supplement: Supplemental Information 3 [file peerj-10-13335-s003.docx]

| Gene | Polymorphism | Author | Year | population | Sample size | | MAF(%)  (controls) | Influence on | References |  |
| --- | --- | --- | --- | --- | --- | --- | --- | --- | --- | --- |
|  |  |  |  |  | cases | controls |  |  |  |  |
| TLR5 | rs5744174  (T/C) | Katrinli et al. | 2018 | Turkish | 131 | 168 | 42.90 | Susceptibility to HBV infection | ^[70]^ |  |
|  |  | Wu et al. | 2012 | Taiwanese | 278 | - | - | HBV clearance | ^[64]^ |  |
|  |  | Cao et al. | 2017 | Chinese | 636 | 273 | 21.80 | The progression of HBV-related liver disease | ^[71]^ |  |
| TLR7 | rs179009  (A/G) | Zhu et al. | 2017 | Chinese | 612 | 293 | Male：14.40  Female：13.30 | The risk and progression of HBV-related liver disease (chronic hepatitis B, LC and HCC) | ^[73]^ |  |
|  | rs179009-rs179010-  rs2074109 haplotype |  |  |  |  |  | - | Susceptibility to HBV infection and the progression of HBV-related liver disease |  |  |
| Abbreviations: MAF: minor allele frequency; LC: [liver](javascript:;) [cirrhosis](javascript:;); HCC: [hepatocellular](javascript:;) [carcinoma](javascript:;). | | | | | | | | | | |
