# Supplementary material for: Association of toll-like receptors single nucleotide polymorphisms with HBV and HCV infection: research status
Source: PeerJ. 2022 Apr 19;10:e13335. doi: 10.7717/peerj.13335 (PMC9029363; doi:10.7717/peerj.13335)
Supplement: Supplemental Information 4 [file peerj-10-13335-s004.docx]

| Polymorphism | Author | Year | population | Sample size | | MAF(%)  (controls) | Influence on | References |
| --- | --- | --- | --- | --- | --- | --- | --- | --- |
|  |  |  |  | cases | controls |  |  |  |
| rs187084  (A/G) | Chihab et al. | 2019 | Moroccan | 239 | 133 | 32.33 | The progression of HBV-related liver disease | ^[74]^ |
| rs5743836  (A/G) | Chihab et al. | 2019 | Moroccan | 239 | 133 | 14.40 | HBV DNA load | ^[74]^ |
|  | Wu et al. | 2012 | Taiwanese | 278 | - | - | HBV clearance | ^[64]^ |
| rs352140  (C/T)  (G/A) | He et al. | 2015 | Chinese Han | 1191 | 273 | - | Susceptibility to HBV infection | ^[76]^ |
|  | Gao et al. | 2015 | Chinese | 51 | 348 | 42.20 | Susceptibility to HBV intrauterine transmission | ^[43]^ |
| Abbreviation: MAF: minor allele frequency. | | | | | | | | |
