# Supplementary material for: Association of toll-like receptors single nucleotide polymorphisms with HBV and HCV infection: research status
Source: PeerJ. 2022 Apr 19;10:e13335. doi: 10.7717/peerj.13335 (PMC9029363; doi:10.7717/peerj.13335)
Supplement: Supplemental Information 5 [file peerj-10-13335-s005.docx]

| Polymorphism | Author | Year | population | Sample size | | MAF(%)  (controls) | Influence on | References |
| --- | --- | --- | --- | --- | --- | --- | --- | --- |
|  |  |  |  | cases | controls |  |  |  |
| rs13105517  (G/A) | Neamatallah et al. | 2020 | Egyptian | 1680 | 1615 | 11.00 | The risk of chronic hepatitis C | ^[77]^ |
| rs3804099  (T/C) | Neamatallah et al. | 2020 | Egyptian | 1680 | 1615 | 27.00 | The risk of HCV-related HCC | ^[77]^ |
| rs1898830-rs1816702-rs13105517 - rs3804099 haplotype |  |  |  |  |  | - | The risk of HCV-related HCC |  |
| -196 to -174 del/ins | Nischalke et al. | 2012 | German | 381 | 347 | 15.25 | The risk of HCV-related HCC; HCV viral load | ^[78]^ |
| Abbreviations: MAF: minor allele frequency; HCC: [hepatocellular](javascript:;) [carcinoma](javascript:;). | | | | | | | | |
