# Supplementary material for: Association of toll-like receptors single nucleotide polymorphisms with HBV and HCV infection: research status
Source: PeerJ. 2022 Apr 19;10:e13335. doi: 10.7717/peerj.13335 (PMC9029363; doi:10.7717/peerj.13335)
Supplement: Supplemental Information 6 [file peerj-10-13335-s006.docx]

| Polymorphism | Author | Year | population | Sample size | | MAF(%)  (controls) | Influence on | References |
| --- | --- | --- | --- | --- | --- | --- | --- | --- |
|  |  |  |  | cases | controls |  |  |  |
| rs3853839  (C/G) | El-Bendary et al. | 2018 | Egyptian | 1908 | 1460 | - | Susceptibility to HCV infection | ^[79, 103]^ |
| rs179008  (A/T) | Fakhir et al | 2017 | Moroccan | 505 | 138 | - | Hepatitis C outcomes and liver disease progression | ^[78, 79]^ |
| rs179009 (A/G) | Yue et al. | 2014 | Chinese Han | 754 | 1013 | Male：17.00  Female：16.70 | Susceptibility to HCV infection | ^[105, 106]^ |
|  | Wei et al. | 2014 | Chinese Han | 150 | 161 | Male：29.07  Female：41.33 | Susceptibility to HCV infection | ^[107]^ |
| rs179016  (G/C) | Xue et al. | 2015 | Chinese | 1176 | 1107 | 13.03 | HCV clearance | ^[108]^ |
| rs1634323  (A/G) | Xue et al. | 2015 | Chinese | 1176 | 1107 | 3.86 | Susceptibility to HCV infection | ^[108]^ |
| Abbreviation: MAF: minor allele frequency. | | | | | | | | |
