# Supplementary material for: Association of toll-like receptors single nucleotide polymorphisms with HBV and HCV infection: research status
Source: PeerJ. 2022 Apr 19;10:e13335. doi: 10.7717/peerj.13335 (PMC9029363; doi:10.7717/peerj.13335)
Supplement: Supplemental Information 7 [file peerj-10-13335-s007.docx]

| Gene | Polymorphism | Author | Year | population | Sample size | | MAF(%)  (controls) | Influence on | References |
| --- | --- | --- | --- | --- | --- | --- | --- | --- | --- |
|  |  |  |  |  | cases | controls |  |  |  |
| TLR8 | rs3764879  (G/C) | El-Bendary et al. | 2018 | Egyptian | 1908 | 1460 | - | Susceptibility to HCV infection | ^[79, 106]^ |
|  | rs3764880  (A/G) | El-Bendary et al. | 2018 | Egyptian | 1908 | 1460 | - | Susceptibility to HCV infection; HCV clearance | ^[79, 106]^ |
|  | rs1013151  (C/T) | Fernández-Rodríguez et al. | 2015 | European descent | 220 | - | - | Progression of chronic hepatitis C in HIV/HCV co-infected patients | ^[112]^ |
|  | rs5744069  (G/T) | Fernández-Rodríguez et al. | 2015 | European descent | 220 | - | - | Progression of chronic hepatitis C in HIV/HCV co-infected patients | ^[112]^ |
| TLR9 | rs5743836  (T/C) | Hamdy et al. | 2018 | Egyptian | 281 | 265 | 12.35 | Susceptibility to HCV infection | ^[13, 89, 113]^ |
|  | rs352140  (G/A) | Hamdy et al. | 2018 | Egyptian | 281 | 265 | 48.20 | Susceptibility to HCV infection | ^[13]^ |
|  |  | Valverde-Villega et al. | 2017 | Brazilian | 374 | 415 | - | Susceptibility to HCV/HIV co-infection | ^[114]^ |
|  |  | Youssef et al. | 2017 | Egyptian | 120 | - | - | HCV-related liver fibrosis and cirrhosis | ^[115]^ |
|  |  | Clausen et al. | 2014 | Caucasian | 308 | 216 | 46.88 | The outcome of HCV infection | ^[116]^ |
|  | rs187084  （T/C） | Fischer et al. | 2017 | Caucasian | 494 | 1057 | 41.5 | HCV clearance | ^[61]^ |
| Abbreviation: MAF: minor allele frequency. | | | | | | | | | |
